# Causes of Death in Long-Term Nasopharyngeal Carcinoma Survivors

**DOI:** 10.3389/fpubh.2022.912843

**Published:** 2022-07-07

**Authors:** Shi-Ping Yang, Ming-Yue Rao, Qing-Shuang Chen, Ping Zhou, Chen-Lu Lian, San-Gang Wu

**Affiliations:** ^1^Department of Radiation Oncology, Hainan General Hospital (Hainan Affiliated Hospital of Hainan Medical University), Haikou, China; ^2^Department of Radiology, The First Affiliated Hospital of Xiamen University, School of Medicine, Xiamen University, Xiamen, China; ^3^Department of Ultrasound, The First Affiliated Hospital of Xiamen University, School of Medicine, Xiamen University, Xiamen, China; ^4^Department of Radiation Oncology, Xiamen Cancer Center, Xiamen Key Laboratory of Radiation Oncology, The First Affiliated Hospital of Xiamen University, School of Medicine, Xiamen University, Xiamen, China

**Keywords:** nasopharyngeal carcinoma, long-term survival, survivors, cause of death, SEER

## Abstract

**Purpose:**

To assess the causes of death (COD) and long-term survival after nasopharyngeal carcinoma (NPC) diagnosis.

**Methods:**

Using linked data from the Surveillance, Epidemiology, and End Results program, patients with NPC diagnosed from 1990 to 2010 and followed up >5 years were identified. Chi-squared test, the Kaplan–Meier method, and the Cox proportional hazard model were used for analyses.

**Results:**

Among the 3,036 long-term NPC survivors, 1,432 survived for >5–10 years and 1,604 survived for >10 years. The most common COD was primary NPC (36.9%), followed by other causes (28.7%), other cancers (15.3%), cardiac disease (12.9%), and non-malignant pulmonary disease (6.2%). With a median follow-up of 125 months, deaths from NPC decreased with increasing time from diagnosis, while death because of cardiac disease and other causes increased. In those aged <50 years, death due to NPC remained the main COD over time, while cardiopulmonary disease-related death was the leading COD in patients aged ≥50 years. In White patients, death due to NPC decreased, and death due to cardiac disease increased over time. Death from NPC remained significant in Black and Asian patients even 15 years after the diagnosis of NPC, while death due to cardiac disease significantly increased after 9 years of diagnosis in Black patients. Multivariate analyses showed that the independent factors associated with inferior NPC-specific survival were older age, Asians, American Indian/Alaska Native, regional stage, distant stage, and diagnosis in the early years.

**Conclusions:**

The probability of death from primary NPC remains significant even 15 years after the NPC diagnosis. Our study advocates continued surveillance for NPC survivors beyond the traditional 5 years. Individualized follow-up strategies are required for patients with NPC of different ages and races.

## Introduction

Nasopharyngeal carcinoma (NPC) is an epithelial carcinoma with distinct racial and geographical distribution. It has been endemic in East and Southeast Asia with incidence rates can be more than 20 per 100,000 person-years, while, in most Western countries, the overall incidence of NPC is <1 per 100,000 person-years (1, 2). Due to the insidious characteristics of NPC, 70–80% of patients were diagnosed with locally advanced disease (3). With the development of radiotherapy techniques (4, 5), chemotherapy drugs (6, 7), and immunotherapy (8, 9), the 5-year overall survival rate of NPC has reached ~85% (10). However, there are still ~20–30% of patients that will develop distant metastasis after comprehensive treatment, especially in those with an advanced nodal stage and high Epstein-Barr virus (EBV) DNA levels (11, 12).

Several long-term studies have investigated the malignant causes of NPC-specific mortality (13–15). However, the information on the nonmalignant causes of death (COD) remains limited in long-term NPC survivors, which may be a driver for guiding treatment strategies and long-term follow-up to address any preventable treatment-related adverse events. Databases based on high-quality population-based cohorts have important guiding value for investigating long-term COD in patients with cancer. However, such studies on NPC are yet to be performed. In this study, we aimed to assess the COD in long-term NPC survivors using a population-based cohort to provide initial insights to guide long-term follow-up strategies.

## Materials and Methods

### Patients

We identified NPC data using the Surveillance, Epidemiology and End Results (SEER) program, a public cancer database that includes de-identified information from population-based cancer registry data in the United States (US) (16). We included patients with primary NPC who received radiotherapy from the years of 1990 to 2010 and survived >60 months. The patients without sufficient survival data and who received non-beam radiation were excluded. The SEER program includes de-identified patient information and is, therefore, exempt from the approval process of the Institutional Review Board.

### Measures

We included the following data in the analysis: age, gender, years of diagnosis, race, histology, SEER historic stage, American Joint Committee on Cancer (AJCC). Tumor Node Metastasis (TNM) stage, chemotherapy use, and COD. The classification of races was divided into White, Black, Asian, and American Indian/Alaska Native. We classified histology into keratinizing squamous cell carcinoma (SCC) (codes: 8070 and 8071) (WHO type I), differentiated non-keratinizing SCC (codes: 8072 and 8073) (WHO type II), undifferentiated non-keratinizing SCC (codes: 8020, 8021, and 8082) (WHO type III), and others. COD was defined as deaths from primary NPC, other cancers, cardiac-related diseases, pulmonary-related diseases, and other causes. We used a simplified version of the SEER historic stage (localized, regional, and distant) to define the stage of patients with NPC because the TNM staging was not available in the SEER database before 2004. The 6th edition of the AJCC TNM staging system was used for those diagnosed between 2004 and 2010. The distribution of COD for long-term NPC survivors was analyzed according to the following latency period to guide the optimal long-term follow-up strategies: 5–7 years, 7–9 years, 9–11 years, 11–13 years, 13–15 years, and >15 years. In the current analysis, NPC-specific survival (NPCSS) was the primary endpoint and was defined as the time from the diagnosis of NPC to death from NPC.

### Statistical Analysis

Chi-square tests were conducted to compare the baseline characteristics between patients who survived >5–10 years and >10 years. Kaplan–Meier analysis and log-rank test were conducted for survival comparisons. A multivariate Cox proportional hazards model was used to evaluate the independent prognostic factors related to NPCSS. All statistical analyses were performed using SPSS v22.0 (IBM Corporation, Armonk, NY, USA) and MedCalc Statistical Software version 18.2.1 (MedCalc Software bvba, Ostend, Belgium), with a *P*-value < 0.05, indicating statistical significance. The histograms presenting the proportion of different COD over time were produced by Microsoft Excel.

## Results

### Baseline Characteristics

**Figure 1:**
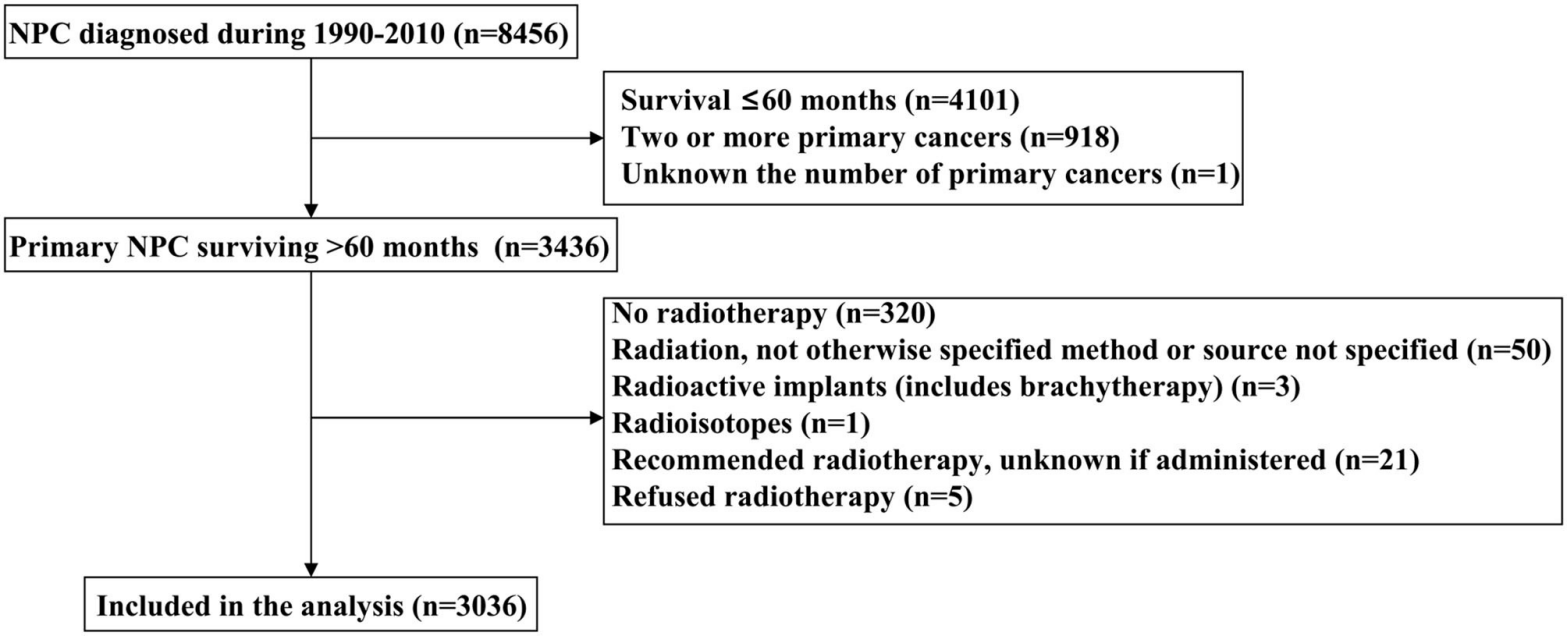
A flow diagram of the study cohort.

A total of 3,036 long-term NPC survivors were included in the current study (Table 1). The patient selection flowchart has been listed in Figure 1. The majority of cases were male (*n* = 2,109, 69.5%), Asians (*n* = 1,491, 49.%), regional stage (*n* = 1,897, 62.5%), and receipt of chemotherapy (*n* = 2,360, 77.7%). Among Asians, 50.% of patients with NPC were Chinese. In patients with available histology, 854 (23.6%), 520 (23.6%), and 834 (37.8%) had keratinizing SCC, differentiated non-keratinizing SCC, and undifferentiated non-keratinizing SCC, respectively. Among patients diagnosed between 2004 and 2010 (*n* = 1,460), 1,303 had available TNM staging data, including 141 (10.8%), 375 (28.8%), 429 (32.9%), and 358 (27.5%) had Stages I, II, III, and IV diseases, respectively. In the entire cohort, 1,432 patients survived >5–10 years and 1,604 patients survived >10 years. The patients with younger age (*p* < 0.001), diagnosis between 1990 and 1999 (*p* < 0.001), Asians (*p* < 0.001), undifferentiated non-keratinizing SCC (*p* < 0.001), localized and regional stages (*p* < 0.001), and no receipt of chemotherapy were more likely to survive >10 years (Table 1).

**Table 1:** Baseline characteristics in long-term nasopharyngeal carcinoma survivors.

**Variables**	***n***	**Patients surviving >5–10 years (%)**	**Patients surviving >10 years (%)**	***P***
**Age (years)**				
<50	1,603	629 (43.9)	974 (60.7)	<0.001
50–64	1,020	538 (37.6)	482 (30.0)	
≥65	413	265 (18.5)	148 (9.3)	
**Years of diagnosis**				
1990–1999	866	217 (15.2)	649 (40.5)	<0.001
2000–2010	2,170	1,215 (84.8)	955 (59.5)	
**Gender**				
Male	2,109	990 (69.1)	1,119 (69.8)	0.707
Female	927	442 (30.9)	485 (30.2)	
**Race**				
White	1,234	628 (43.9)	606 (37.8)	<0.001
Black	268	138 (9.6)	130 (8.1)	
Asian	1,491	640 (44.7)	851 (53.1)	
American Indian/Alaska Native	43	26 (1.8)	17 (1.1)	
**Histology**				
WHO type I	854	415 (29.0)	439 (27.4)	<0.001
WHO type II	520	285 (19.9)	235 (14.7)	
WHO type III	834	336 (23.5)	498 (31.0)	
Other	828	396 (27.7)	432 (26.9)	
**SEER stage**				
Localized	343	148 (10.3)	195 (12.2)	<0.001
Regional	1,897	788 (55.0)	1,109 (69.1)	
Distant	687	455 (31.8)	232 (14.5)	
Other	109	41 (2.9)	68 (4.2)	
**Chemotherapy**				
No	676	261 (18.2)	415 (25.9)	<0.001
Yes	2,360	1,171 (81.8)	1,189 (74.1)	

### COD in Long-Term NPC Survivors

With a median follow-up of 125 months (range, 61–323 months), a total of 885 deaths were observed, and 327 of them died from NPC. The most common COD was primary NPC (*n* = 327, 36.9), followed by other causes (*n* = 254, 28.7%), other cancers (*n* = 135, 15.3%), cardiac-related disease (*n* = 114, 12.9%), and non-malignant pulmonary disease (NPD) (*n* = 55, 6.2%). The patterns of the COD vary among patients with different survival times. Among patients surviving >5–10 years, 44.1% (*n* = 255) died due to primary NPC, 24.2% (*n* = 140) due to other causes, 16.8% (*n* = 97) due to other cancers, 10.6% (*n* = 61) due to cardiac disease, and 4.3% (*n* = 25) due to NPD. For patients surviving >10 years, 37.1% (*n* = 114) died due to other causes, 23.5% (*n* = 72) due to primary NPC, 17.3% (*n* = 53) due to cardiac disease, 12.4% (*n* = 38) due to other cancers, and 9.8% (*n* = 30) due to NPD.

**Figure 2:**
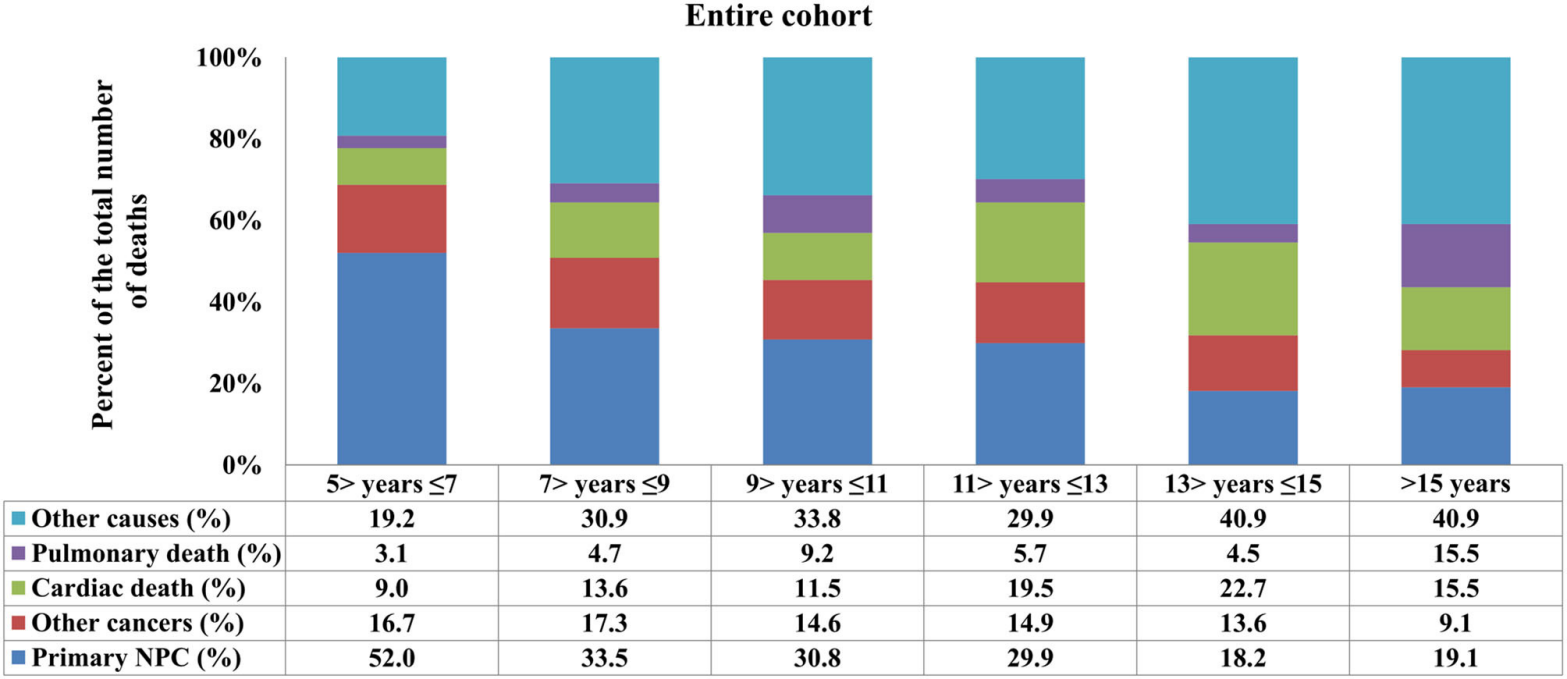
Causes of death across different time periods for long-term nasopharyngeal carcinoma survivors.

**Figure 3:**
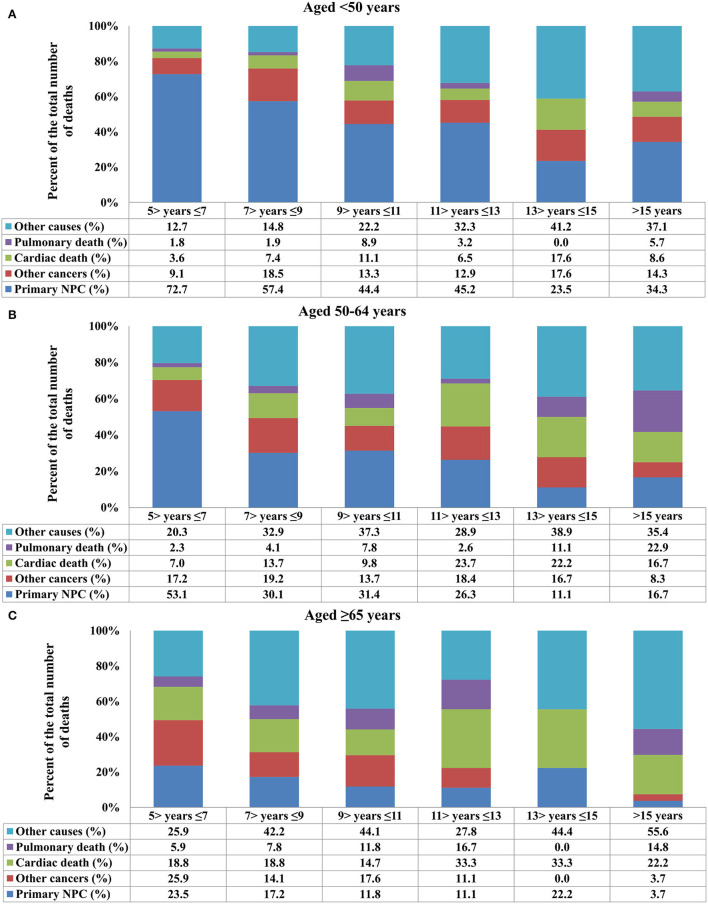
Causes of death after stratification by age at diagnosis for long-term nasopharyngeal carcinoma survivors over time [**(A)** aged <50 years; **(B)** aged 50–64 years; **(C)** ≥65 years].

**Figure 4:**
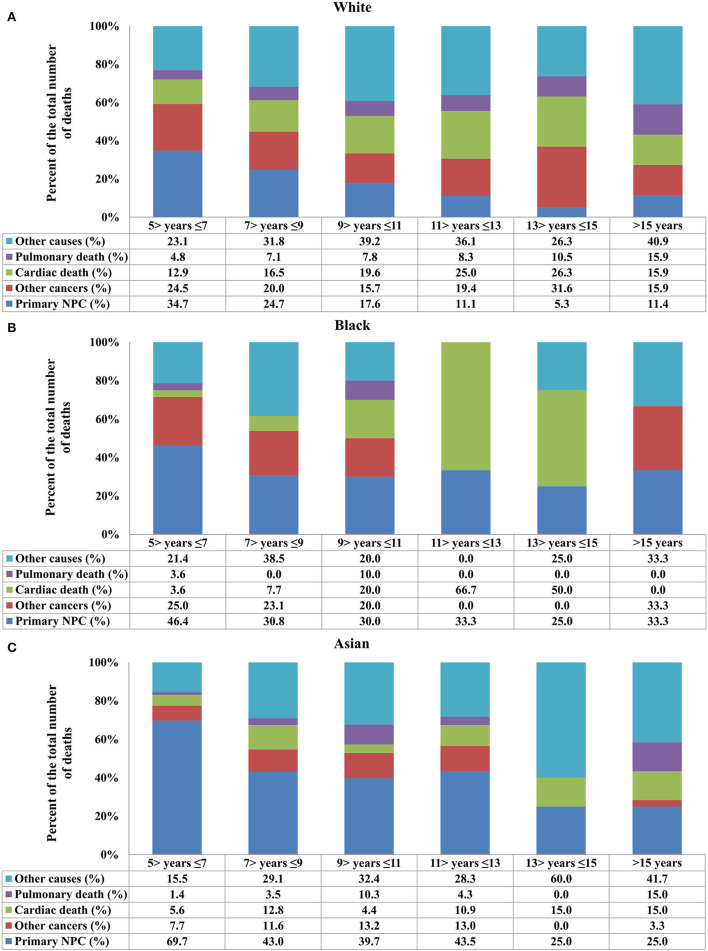
Causes of death after stratification by race for long-term nasopharyngeal carcinoma survivors over time [**(A)** White; **(B)** Black; **(C)** Asian].

In the entire cohort, death from NPC decreased with increasing time from diagnosis, while death because of cardiac disease and other causes increased (Figure 2). The COD after stratification by years of survival according to age and race is listed in Figures 3, 4. In patients aged <50 years, death due to NPC remained the main COD over time, and death from NPC was still significant even > 15 years after diagnosis of NPC (Figure 3A). For patients aged 50–64 years, NPC-related deaths gradually decreased over time, and NPD-related deaths peaked at >15 years (Figure 3B). In addition, in those aged ≥65 years, death due to cardiac disease significantly increased after 11 years of diagnosis and was the predominant COD for whom the exact COD was known (Figure 3C).

Regarding race, death due to NPC decreased in White patients, and death due to cardiac disease increased (Figure 4A). In Black patients, death due to NPC remained significant even 15 years after the diagnosis of NPC, and cardiac disease-related death significantly increased after 9 years of diagnosis (Figure 4B). In Asian patients, NPC remained the main COD over time, and cardiac death risk was no higher than in White and Black patients (Figure 4C). The distribution of COD was similar between Chinese and other Asians (all *p* > 0.05).

### Survival Outcomes and Prognostic Analyses

**Figure 5:**
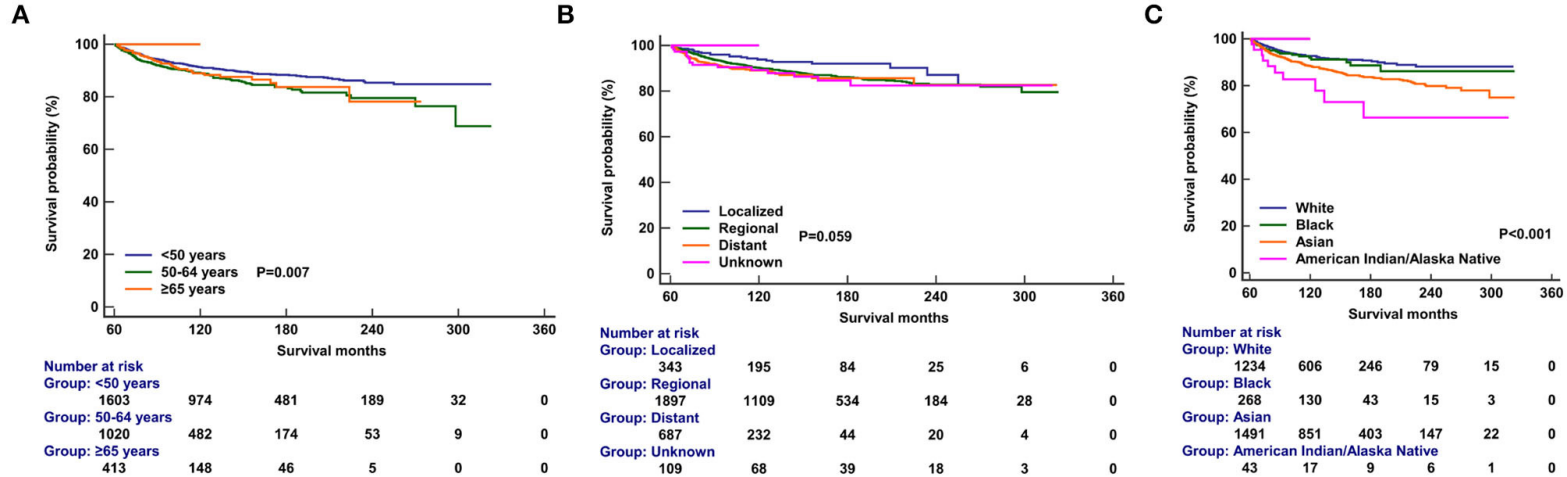
Comparison of nasopharyngeal carcinoma-specific survival after stratification by age at diagnosis **(A)**, race **(B)**, and SEER stage **(C)**.

Multivariate analyses were conducted to evaluate the independent factors associated with NPCSS (Table 2). In the NPC survivors, independent factors associated with inferior NPCSS included older age [aged 50–64 years vs. aged <50 years, the hazard ratio (HR) 1.559, 95% confidence interval (CI), 1.230–1.977, *p* < 0.001], Asian (Asian vs. White, HR, 1.757, 95% CI, 1.368-2.257, *p* < 0.001), American Indian/Alaska Native (American Indian/Alaska Native vs. White, HR, 3.072, 95% CI, 1.592–5.927, *p* = 0.001), the regional stage (the regional stage vs. the localized stage, HR, 1.721, 95% CI, 1.127–2.626, *p* = 0.012), the distant stage (the distant stage vs. the localized stage, HR, 2.226, 95% CI, 1.389–3.565, *p* = 0.001), and diagnosed between 1990 and 1999 (diagnosed between 1990 and 1999 vs. diagnosed between 2000 and 2010, HR, 1.276, 95% CI, 1.00–21.627, *p* = 0.048) (Table 2). The survival curves according to age at diagnosis (Figure 5A), race (Figure 5B), and SEER stage (Figure 5C) are listed in Figure 4. In those diagnosed between 2004 and 2010, the AJCC stage was the only independent prognostic factor associated with NPCSS (Table 3).

**Table 2:** Multivariate survival analysis for nasopharyngeal carcinoma-specific survival in long-term survivors in the entire cohort.

**Variables**	**HR**	**95%CI**	**P**
**Age (years)**			
<50	1		
50–64	1.559	1.230–1.977	<0.001
≥65	1.414	0.994–2.012	0.054
**Years of diagnosis**			
1990–1999	1		
2000–2010	0.783	0.615–0.998	0.480
**Gender**			
Male	1		
Female	1.150	0.913–1.448	0.236
**Race**			
White	1		
Black	1.271	0.801–2.015	0.309
Asians	1.757	1.368–2.257	<0.001
American Indian/Alaska Native	3.072	1.592–5.927	0.001
**Histology**			
WHO type I	1		
WHO type II	1.075	0.766–1.507	0.677
WHO type III	0.931	0.690–1.257	0.642
Other	1.055	0.785–1.421	0.717
**SEER stage**			
Localized	1		
Regional	1.721	1.127–2.626	0.012
Distant	2.226	1.389–3.565	0.001
Other	1.851	0.969–3.536	0.062
**Chemotherapy**			
No	1		
Yes	0.902	0.681–1.194	0.471

**Table 3:** Multivariate survival analysis for nasopharyngeal carcinoma-specific survival in long-term survivors diagnosed between 2004 and 2010.

**Variables**	**HR**	**95%CI**	**P**
**Age (years)**			
<50	1		
50–64	1.215	0.761–1.938	0.415
≥65	0.913	0.421–1.982	0.818
**Gender**			
Male	1		
Female	1.093	0.685–1.746	0.709
**Race**			
White	1		
Black	0.545	0.226–1.313	0.176
Asiane	0.957	0.598–1.532	0.856
American Indian/Alaska Native	2.377	0.562–10.009	0.238
**Histology**			
WHO type I	1		
WHO type II	1.365	0.694–2.688	0.367
WHO type III	1.661	0.869–3.175	0.125
Other	1.602	0.836–3.070	0.155
**Clinical stage**			
I	1		
II	1.400	0.465–4.217	0.550
III	2.491	0.876–7.087	0.087
IV	3.746	1.329–10.562	0.012
**Chemotherapy**			
No	1		
Yes	0.980	0.419–2.294	0.963

## Discussion

In the current study, we used a population-based cohort to analyze the COD in long-term NPC survivors (>5 years). Our study was the first to tackle this issue, and our findings indicated that, with increased follow-up time after NPC diagnosis, death due to primary NPC or other second cancers decreased; while death due to non-cancer-related causes increased. In addition, the probability of death from primary NPC remained significant (~20%) even 15 years after NPC diagnosis.

In the current European Society for Medical Oncology guidelines, Magnetic Resonance Imaging should be used one time every 6 months for at least 3 years after treatment, the nasal examination should be administrated for the first 5 years, and EBV-DNA should be evaluated at least every year (17). The Chinese Society of Clinical Oncology guidelines for NPC have similar recommendations, with an additional recommendation for annual review after 5 years of diagnosis (18). In this study, we found that, although the mortality from primary NPC declined, it did not drop to zero even 15 years after NPC diagnosis. This signifies that neither 5-year nor 10-year long-term survival signifies a cure of NPC, and long-term surveillance for NPC survivors is required. Several population-based studies have also shown similar findings in other cancers, including lung (19), bladder (20), and head and neck cancers (21). Our study suggests that routine follow-up care for NPC survivors should be expanded for a long period.

Similar to other head and neck cancers (22), NPC survivors also faced an increased risk of death from any cause. The results from a non-endemic area showed that the risk of differentiated NPC was significantly associated with tobacco smoking like other head and neck cancers, while smoking had limited influence on the risk of undifferentiated NPC (23). However, a meta-analysis showed that tobacco smoking increased NPC risk in the endemic areas (24). Therefore, in addition to the additional risk posed by NPC, the increased mortality likely reflects tobacco use among long-term NPC survivors. Tobacco smoking is a common risk factor in NPC, and it is also linked to cardiopulmonary disease and several other cancers. However, estimates of excess mortality from each cause in long-term NPC survivors should include comparisons with peers of similar age at diagnosis, race/ethnicity, and history of tobacco use.

In our previous study, we found that younger NPC patients were more likely to develop the locally advanced disease than their older counterparts (25). Several studies including ours have also confirmed that patients with younger age have a better prognosis than older NPC (25–27). However, there are no relevant studies on the effects of different age distributions on long-term COD of NPC. In our study, NPC-related death remained the main COD over time in patients aged <50 years, and NPC-related deaths gradually decreased over time, whereas non-cancer-related causes increased over time in those aged ≥50 years. Therefore, cardiopulmonary disease assessment is required for older patients with a long-term follow-up, while primary NPC should remain a primary consideration during the long-term follow-up of younger survivors.

In the current study, we also found that long-term NPC African Americans survivors had a higher risk for cardiac death than Asian and White patients. This may be partly explained by the fact that African Americans have a higher overall risk of death from heart disease compared with Asians and Whites (28). However, NPC remained the main cause of death during the follow-up period of 5–13 years in Asian patients, which was higher than Whites and African Americans. This can be explained by the different incidence rates of NPC by race and ethnicity. Therefore, for NPC patients undergoing a long-term follow-up, optimal healthcare needs to be formulated according to different ethnic groups.

In our study, independent factors associated with inferior NPCSS in multivariate analysis included older age, Asians, American Indian/Alaska Native, the regional stage, the distant stage, and diagnosed in early years. American Indians/Alaskan Natives had the worse NPCSS compared to White Americans, which was similar to the previous study (29). Surprisingly, we found that the long-term NPCSS of Asians was significantly worse than that of Whites. The reason for this result remains unclear. In several previous SEER studies, Chinese or other Asians had better survival outcomes than other races/ethnicity (30–32). However, none of them assessed the long-term survival outcomes of the patients. Keratinizing SCC is the predominant subtype in Whites, while the undifferentiated non-keratinizing SCC type is predominant in Asians. In our previous study, patients with keratinizing SCC reached a peak mortality in the 1st year after diagnosis, and the mortality rate decreased significantly in the 5th year after diagnosis. However, patients with undifferentiated non-keratinizing SCC had a peak mortality in both the 2nd and 6th years of diagnosis (33). Therefore, the higher long-term mortality risk in Asian patients than in White patients may be related to the peak mortality across different histological subtypes.

Several inherent limitations should be acknowledged in this study. First, the lack of information on baseline comorbidities in the SEER database may have influenced the patterns of COD in this population. Second, unified AJCC staging cannot be used to assess the survival of patients due to the long time width of the included patients, which may confound the prognostic evaluation of patients. Third, it is difficult to determine whether a patient with recurrent NPC is a true recurrence or a new primary NPC. Moreover, the prognosis of NPC is also connected to EBV DNA. However, the status of EBV DNA is not included in the current SEER database. Finally, the data for this study were generated from the SEER program in the US. Therefore, our findings are not representative of the entire patient population, especially those from NPC endemic areas.

## Conclusions

In conclusion, our study suggests that the probability of death from primary NPC remains significant even 15 years after the NPC diagnosis. The surveillance for NPC survivors should be continued beyond the traditional 5 years. Individualized follow-up strategies are required for patients with NPC of different ages and races.

## Data Availability Statement

The raw data supporting the conclusions of this article will be made available by the authors, without undue reservation.

## Ethics Statement

Ethical review and approval was not required for the study on human participants in accordance with the local legislation and institutional requirements. Written informed consent for participation was not required for this study in accordance with the national legislation and the institutional requirements.

## Author Contributions

S-PY, M-YR, and Q-SC drafted the manuscript. S-GW acquired the datasets, conceived the study, and conducted the statistical analyses. S-GW, PZ, and C-LL participated in the study design. All authors read and approved the final manuscript.

## Funding

This work was partly supported by the Natural Science Foundation of Fujian Province (No. 2020J011220) and the Key Medical and Health Projects in Xiamen (No. 3502Z20209002).

## Conflict of Interest

The authors declare that the research was conducted in the absence of any commercial or financial relationships that could be construed as a potential conflict of interest.

## Publisher's Note

All claims expressed in this article are solely those of the authors and do not necessarily represent those of their affiliated organizations, or those of the publisher, the editors and the reviewers. Any product that may be evaluated in this article, or claim that may be made by its manufacturer, is not guaranteed or endorsed by the publisher.
